# Integration of handcrafted and deep-level features to improve skin disease detection

**DOI:** 10.3389/frai.2026.1732440

**Published:** 2026-03-20

**Authors:** Ranjana Kedar, Manoj Kumar Rajagopal

**Affiliations:** School of Electronics Engineering, Vellore Institute of Technology, Chennai, India

**Keywords:** Explainable Artificial Intelligence (XAI), Gray Level Co-occurrence Matrix features (GLCM), Histogram of Oriented Gradient (HOG) features, improved Local Binary Pattern (ILBP), Skin Disease Detection Network (SDNet)

## Abstract

As per the World Health Organization, skin disorders are some of the most common health issues, constituting a significant source of non-fatal disease burden and greatly affecting both quality of life and healthcare systems. Melanoma skin cancer is the most fatal disease in the world, and nearly 60,000 people died due to melanoma in 2022. Skin disorders affect millions of people globally and pose a serious threat to public health. These disorders can affect the skin's composition, function, and appearance. To detect and predict skin diseases, a comprehensive physical examination, a review of the patient's medical history, and appropriate laboratory testing are required. Noise, blur, uneven illumination, inadequate feature representation, and overlapping features between distinct skin disorders all reduce the efficacy of skin disease identification. The Skin Disease Detection Network (SDNet), a deep convolutional neural network-based two-way feature depiction system for multiclass skin disease identification, is presented in this study. To depict hierarchical features in skin images, the SDNet employs two parallel arms. The first arm uses a 2D CNN in conjunction with pre-processed original images, while the second arm uses a 1D CNN that accepts Gray Level Co-occurrence Matrix features (GLCM), improved Local Binary Pattern (ILBP), and Histogram of Oriented Gradient (HOG) features to depict texture and shape attributes of skin images. The accuracy, recall, precision, and F1-score of SDNet results are evaluated using the DermNet dataset. The proposed SDNet achieves an overall accuracy of 99.1%, a recall of 99.1%, a precision of 98.96%, and an F1-score of 98.95% for the 5-class skin disease detection, demonstrating a notable improvement over the performance of traditional state-of-the-art methods. This study marks a notable step forward in leveraging the reliability of SDNet for precise and efficient skin disease identification through the Explainable Artificial Intelligence (XAI) approach.

## 1 Introduction

The World Health Organization states that skin diseases are the leading cause of illness worldwide. In 2022, there were an expected 1.5 million cases of skin disease, and 60,000 people died from skin melanoma (World Health Organization, 2024). The most common cause of skin illness is excessive sun exposure. Many skin problems have become more dangerous over time. Physical diagnosis can also be challenging because many skin disorders have similar visual characteristics, which makes medical diagnosis and treatment more challenging. Skin illnesses are usually identified using the ABCDE criteria, which consider asymmetry, border, color, dimension, and evolution (Dildar et al., 2021; Javed et al., 2020). Automated Skin Disease Detection (ASDD) is important since a variety of diseases show overlapping symptoms in skin scans. The largest organ in humans is the skin. To diagnose skin disorders, dermatologists have traditionally employed dermatoscopy, biopsy, and ocular examination (Al Mamun and Uddin, 2021; Zhang et al., 2021; Goswami et al., 2020; Haddad and Hameed, 2018). Nowadays, dermatologists' knowledge is the main determinant of the ASDD's effectiveness, which may be limited by their scarcity, lack of comprehension, variations in inter-observer variability, and visibility problems (Diame et al., 2021; Voggu and Rao, 2022). The traditional vision-based ASDD uses skin images to identify diseases. This process involves steps such as pre-processing, extracting features, and classifying data using machine learning (ML) and deep learning (DL) classifiers. However, the visual characteristics of several overlapping disorders, such as low resolution, blurriness, difficulties differentiating skin lesions from surrounding skin, poor contrast, and picture noise, make the results of the current ASDD difficult to understand (Janoria et al., 2021; Hussein and Stephan, 2021). A deep learning-based method for detecting skin diseases is presented in this paper. The following is a paper's summary, which indicates noteworthy contributions:

- Improved contrast, text, and edge-aware filtering (ICTEF) is a new technique for improving skin images. It uses double-stage Gaussian, Wiener, and median filtering to enhance edges, texture, reduce noise, and improve contrast.
- Two-way feature depiction utilizing SDNet, which includes both 2D-CNN and 1D-CNN. The former accepts pre-processed pictures, while the latter accepts enhanced LBP, GLCM, and HOG features for skin disease characterization. Better spatial correlation and connectivity are made possible by the improved LBP, which enhances the description of skin image texture.
- Grad-CAM analysis of SDNet features to improve explainability through deep feature visualizations.
- SDNet's accuracy-based performance evaluation on the DermNet Dataset

The structure of the remaining article is as follows: The literature evaluation of current DL-based skin disease detection methods is provided in Section 2. Details of the suggested skin detection system are provided in Section 3. The methodology employed in this work is further explained in Sections 4, and Section 5 discusses the outcomes of several approaches for skin disease diagnosis using SDNet. Section 6 concludes the suggested illness detection paradigm and describes potential future developments for improved detection outcomes.

## 2 Related work

In recent decades, DL has revolutionized machine learning for a range of signal processing applications. Speech recognition (Haddad and Hameed, 2018) pattern identification (Diame et al., 2021), and bioinformatics (Voggu and Rao, 2022) all make use of DL. DL systems perform better than other ML techniques in a number of ways. Analysis of data and pre-processing, model construction and development, verification, and interpretation are all used in DL approaches for different purposes. For computer vision, deep convolutional neural networks, or CNNs, are essential. It gathers, identifies, and classifies pictures. CNN is useful for local as well as global data collecting and analysis since it uses curves and edges to construct forms and corners. CNN employs linked hidden layers, convolution, and non-linear pooling (Janoria et al., 2021; Hussein and Stephan, 2021; Li et al., 2021). CNN is dominated by convolution, pooling, and fully connected layers. MobileNetV2, an LSTM skin condition categorization tool, was created by (Kshirsagar et al. 2022). The device's main goal is to identify skin problems effectively. It requires accurate estimations and high-quality unstructured data. Global fitness is at risk due to skin problems. Physicians are concerned about skin issues. Poor diets and pollution are contributing to an increase in skin problems. The most serious signs of skin disease are frequently ignored by people. Most skin diseases are diagnosed and treated with biopsies that have been approved by a doctor. A hybrid approach could take the place of human analysis if it produces positive results. (Burlina et al. 2019) developed a 95% accurate DCNN for acute Lyme disease using a cross-sectional dataset. There is evidence of healthy skin, herpes zoster, and tinea. One large study used cross-sectional imaging datasets to categorize erythema into everyday, non-pathogenic skin disorders, shingles, and tinea corporis. Medical image recognition, segmentation, and classification are successfully accomplished by CNN-inspired deep neural network systems (Nasr-Esfahani et al., 2016). A DCNN was proposed by (Yu et al. 2017) for the detection of melanoma. Segmentation was enhanced with a fully convolutional residual network (FCRN) with 16 residual blocks. The SVM and softmax classifiers were averaged in the suggested data classification technique. Without segmentation, the accuracy of melanoma classification was 82.8% and 85.5%. An ImageNet-trainable deep neural network (DNN) multi-scale CNN (MSCNN) was proposed by DeVries and Ramachandram (DeVries and Ramachandram, 2017). On input lesion pictures, Enhanced Inception, version 3, can classify skin disorders at both coarse and fine-scale resolutions. Contextual and lesion shape data were collected on a coarse scale. Skin lesions may be distinguished by finer-scale textual information. (Mahbod et al. 2019) used pre-trained DCNNs to classify skin lesions. ResNet-18, AlexNet, and VGG-16 were trained using deep feature representations. These features were used in SVM training. The suggested classification approach for seborrheic keratosis and melanoma has an AUC of 97.55% and 83.83%, respectively, using the ISIC 2017 dataset. Twelve skin lesions were classified by the deep CNN architecture ResNet-152, which was pre-trained (Mendes and da Silva, 2018). Following training, 29 augmentation times were applied to 3,797 lesion pictures based on variations in illumination and scale. Using the proposed technique, skin lesions linked to hemangioma, pyogenic granuloma, and intraepithelial carcinoma (IC) were classified with an AUC of 0.99. CNN was used by (Dorj et al. 2018) to classify four images of skin lesions.Following feature extraction using a DCNN such as AlexNet and the use of an error-correcting output coding SVM classifier, the suggested approach provides the greatest overall scores for dependability, specificity, and sensitivity. Kalouche's deep CNN consists of three final upgraded layers and five CNN blocks (Kalouche, 2016). Seventy-eight percent of lesion images were recognized as melanoma by VCG-16 models. In skin images, a CNN-based technique detected the edges of skin lesions. One thousand two hundred pictures of healthy skin and 400 pictures of sick skin were used to train the DL model. With 86.67% accuracy, the proposed approach distinguished between images of healthy and sick skin. (Ali and Al-Marzouqi 2017) used LightNet DL and ISIC 2016 to classify cases of Benign Skin Lesion (BSL) and Malignant Skin Lesion (MSL). This model is appropriate for mobile apps because of its reduced parameters. It did well with 81.6% accuracy, 14.9% sensitivity, and 98% specificity. (Esteva et al. 2017) suggested classifying BSL SK lesions, keratinocyte carcinomas, and melanoma using Deep CNN using the ISIC-Dermoscopic Archive. Having 72.1% accuracy and knowledge, it fared better than 21 dermatologists. With a 93.75% accuracy rate, (Mandache et al. 2018) classified melanoma/non-melanoma using DermIS and DermQuest data using a CNN-generated SVM-CNN technique for the feature extraction and a median filter to reduce noise. (Harangi et al. 2018) used the CNN ensembles of AlexNet, VGGNet, and GoogleNet to classify MSL melanoma/nevus/SK on ISIC 2017. The model's AUC was 84.8% and its accuracy was 83.8%. Deployment on low-resource standalone devices is limited by the larger trainable parameters of GoogleNet, VGGNet, and AlexNet. (Mandache et al. 2018) used CNN to classify 40 Full-field optical coherence tomography (FF-OCT) pictures as either BCC or no BCC. The CNN model achieves 95.2% sensitivity, 96.54% specificity, and 95.93% accuracy for feature extraction with 10 layers. CNN was used by (Sabouri and Hosseini 2016) to classify 1,730 lesions on the skin and backgrounds as either MSL or non-diseaseous. The accuracy of this edge recognition model was 86.67%. Edge detection systems are impacted by blur, light fluctuations, and image noise. Hasan et al.'s CNN on ISIC classified BSL and MSL samples (Hasan et al., 2019). In 89.5% of cases, the model successfully detected the ABCD symptoms checklist attributes. Lighting, blur, noise, and image quality all have an impact on system performance. (Rezvantalab et al. 2018) used the HAM10000 and PH2 datasets to classify skin lesions using a deep CNN architecture. DL models performed at least 11% better than highly skilled dermatologists, with ROC AUC ranging from 96.10% to 98.79%. (Nida et al. 2019) classified lipoma, sclerosis, fibroma, and melanoma with 94.8% accuracy, 97.81% sensitivity, 94.17% specificity, and a 95.89% F1 score using region-based CNN and fuzzy C-means (FCM) clustering on the ISIC. This model did well. CNN delivers geographical data but ignores the long-term correlations between picture properties. Using the MED-NODE and ISIC datasets, (Mahecha et al. 2018) proposed a 6-layer deep CNN to classify lesions as BSL or MSL. Despite being 77.50%, CNN accuracy was impacted by image lighting. To determine whether ISIC lesions were melanoma or not, (Attia et al. 2017) employed a hybrid CNN model that included an auto-encoder, decoder, and RNN. With 98% accuracy, 93% Jaccard index, 95% sensitivity, and 94% specificity, the model's performance is superior to the best known state-of-the-art methods. Both trainable parameters and computing complexity are increased in a hybrid model. The model cannot be run on a solitary computer with inadequate resources. (Albahar 2019) detected BSL and MSL cells using a 2-layer CNN and a special ISIC regularizer. The accuracy, sensitivity, and specificity of the suggested regularization method are 97.49%, 98%, and 93.6%, respectively. (Namozov et al. 2018) suggested a CNN-based LeNet with an adaptive activation with a linear features function to detect melanoma, melanocytic nevus, BCC, dermatofibroma, BSL keratosis, and vascular lesions. The model achieved 95.86% accuracy. ISIC data was categorized using Deep CNN BSL/MSL by (Singh and Nwogu 2018). The results of data augmentation for balance were 69% AUC, 81% precision, and 80.3% accuracy. Skin disease detection is significantly enhanced by data augmentation. (Liao et al. 2016) used CNN on several datasets to classify MSL melanoma and complex nevus. The pre-trained and improved BVLC-AlexNet model achieves 70% mean average accuracy because of ImageNet. The pre-trained model based on AlexNet performed badly and required more computation time because of its huge trainable parameter set. Due to the rising incidence of dermatological conditions throughout the world, early and precise skin disease identification has gained importance in healthcare in recent years. Deep understanding and optimization-driven approaches have gained popularity since traditional diagnostic methods are frequently constrained by subjectivity and the difficulty of differentiating between visually identical situations. The EfficientNet Squirrel Search Optimization algorithm (ESSO), a hybrid framework that combines EfficientNet with the Squirrel Search Optimization method, was introduced by (Muthulakshmi et al. 2024). Their approach's 98.62% accuracy demonstrated the promise of fusing deep learning with bio-inspired optimization to improve the categorization of skin diseases and facilitate early therapies. (Pandiarajan et al. 2024) advanced the field of CNNs by developing DeepSkinNet, which integrates systematic pre-processing methods like normalization, augmentation, rotation, and flipping, along with dropout layers to improve resistance to overfitting. Their model, evaluated using datasets from Kaggle and web scraping, demonstrated remarkable effectiveness in diagnosing melanoma and other skin conditions, highlighting the transformative role of CNNs in dermatology. (Alnuaimi et al. 2024) focused on children in Saudi Arabia, where significant exposure to ultraviolet (UV) light presents notable dermatological risks. By employing transfer learning models such as MobileNet and DenseNet121, their strategy achieved classification accuracies of 99.99% for eczema and 97% for psoriasis, showcasing the potential of deep learning in addressing healthcare challenges unique to specific regions. (Malik et al. 2024) created a lightweight Computer-Assisted Diagnosis (CAD) system featuring a CNN with seven convolutional layers. Applied to the ISIC dataset, their model achieved an accuracy of 87.64%, offering a more computationally efficient solution while maintaining high diagnostic accuracy. (Kaushik et al. 2024) investigated the use of DenseNet-121 for detecting melanoma, achieving a 90% accuracy rate on the ISIC 2019 dataset. Their findings emphasize DenseNet's versatility in identifying various lesion types, making it a promising tool for early cancer screening. (Abbas et al. 2024) evaluated multiple CNN architectures, such as Sequential CNN, DenseNet-121, and ResNet-50, using the HAM10000 dataset. By employing image augmentation to equalize class distributions, they reached an accuracy of up to 98%, highlighting the significance of balanced datasets and the choice of architecture in detecting multiple skin diseases. (Vidhyalakshmi and Kanchana 2024) proposed a hybrid flash butterfly optimized convolutional neural network with bidirectional long short-term memory (HFB-CNN-BiLSTM), which merges CNN feature extraction with BiLSTM's sequential learning. Their approach, applied to HAM10000 dermoscopic images, achieved 96.3% accuracy, demonstrating the benefits of combining temporal and spatial features for intricate dermatological classifications. (Vishwakarma et al. 2024) advanced beyond CNNs by creating an optimized vision transformer (ViT) model for detecting psoriasis. This model incorporated CNN features into transformer encoder layers and utilized the Adaptive Rabbit Optimization Algorithm (AROA), attaining an accuracy of 97.7% and an F-Score of 96.5%. This highlights the potential of transformer-based architectures in the field of medical imaging. (Gairola et al. 2025) introduced a multi-feature fusion network (FFN) that combines enhanced single and fusion blocks to improve classification. Their framework, tested on various datasets such as ISIC2016, ISIC2017, and HAM10000, reached up to 92% accuracy, consistently outperforming traditional CNNs. (Shukla et al. 2024) emphasized the importance of human–machine collaboration through a large-scale teledermatology study with over 800 physicians from 39 countries. The findings showed that AI decision support notably increased diagnostic accuracy for both specialists and generalists, although challenges remained in diagnosing conditions on darker skin tones, highlighting both the advantages and limitations of AI-assisted dermatology. Lastly, (Badr et al. 2025) initiated a multi-model deep learning architecture based on Xception and transfer learning for classifying multiple skin diseases. Their model, trained on more than 25,000 images across five categories, achieved 95% accuracy and AUROC values exceeding 99%, surpassing previous methods and providing a comprehensive solution for large-scale dermatological diagnostics. From the comprehensive survey, several research gaps have been identified:

- A detailed examination of transfer learning highlights the need for substantial computational resources and an increased number of trainable parameters in current DL models. This limits the application of these algorithms on devices with limited processing capabilities.
- The sequential DCNN is unable to capture both fine and coarse texture features of images simultaneously, as it uses convolution filters of the same kernel size.
- Traditional DL methods yield inconsistent and less generalizable results when there are changes in an image's size, orientation, and lighting (Ali and Al-Marzouqi, 2017).
- The design of sequential DCNNs limits the distinctiveness of features by increasing intra-class similarity and reducing inter-class variability (Rezvantalab et al., 2018; Nida et al., 2019; Mahecha et al., 2018; Attia et al., 2017; Albahar, 2019).
- There is a lack of explainability in DCNNs, which is necessary to enhance trust and comprehension of the model's decision-making processes for practitioners (Tyagi et al., 2024; Mahajan et al., 2022).

## 3 Proposed work

**Figure 1:**
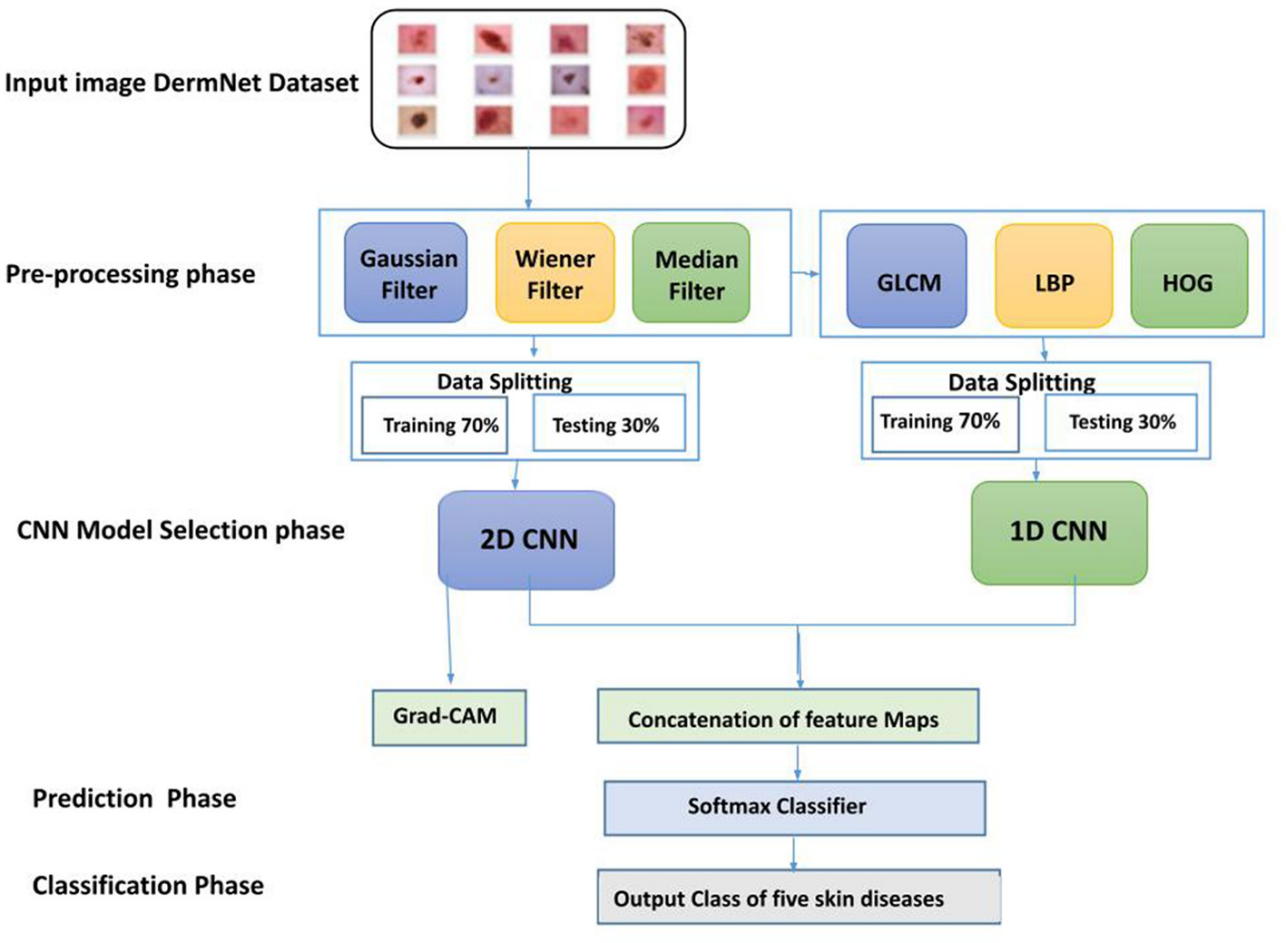
Block diagram of proposed skin disease detection.

The tailored Convolutional Neural Network (CNN) framework created for classifying skin diseases is illustrated in Figure 1. Artificial intelligence, especially CNNs, presents a promising approach to addressing these issues. CNNs are particularly effective in examining skin lesions due to their exceptional capabilities in image recognition and classification. By training these networks with large datasets of skin images, we can create highly precise models that can identify a range of skin conditions. These models can be extremely beneficial for dermatologists, aiding in differential diagnosis and minimizing the chances of misdiagnosis. Additionally, they have the potential to improve access to dermatological care by facilitating remote diagnosis and triage. The proposed system is composed of three components: pre-processing, prediction, and classification. For the experiments, images are obtained from the DermNet collection. To enhance image representation, a multi-stage pre-processing module is introduced, which effectively pre-processes the dataset's images by eliminating noise, boosting contrast, and merging images. For a given input image, the prediction module provides the system's prediction. The deep learning model for the proposed system comprises One-D and Two-D CNNs. The inefficiencies of individual CNN models are addressed by combining the two models, which perform better together than separately.

## 4 Methodology

**Figure 2:**
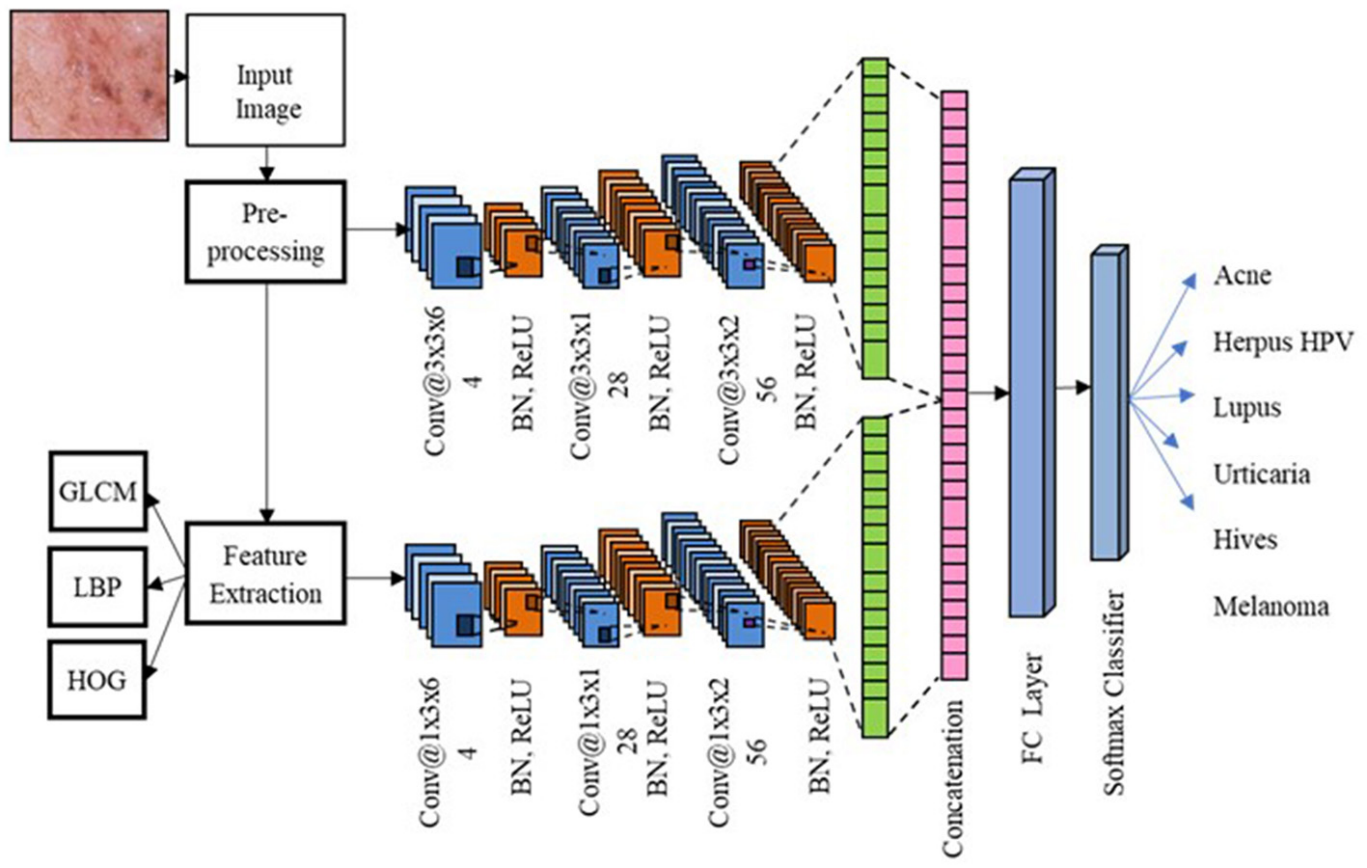
Flow diagram of proposed skin disease detection.

The proposed SDNet utilizes a parallel configuration of 2D-CNN and 1D-CNN to characterize the original skin images and their features, as illustrated in Figure 2. The 2D-CNN processes the original skin images and provides hierarchical features to depict the disease's effect on skin texture. Additionally, the 1D-CNN processes handcrafted features, such as GLCM, LBP, and HOG features, of the skin images that describe the global texture, local texture, and shape attributes of these images. The features from the 1D-CNN and 2D-CNN are combined and fed into the fully connected (FC) layer. Finally, a softmax classifier (SMC) is employed to classify skin images into five skin diseases.

### 4.1 Pre-processing

**Figure 3:**
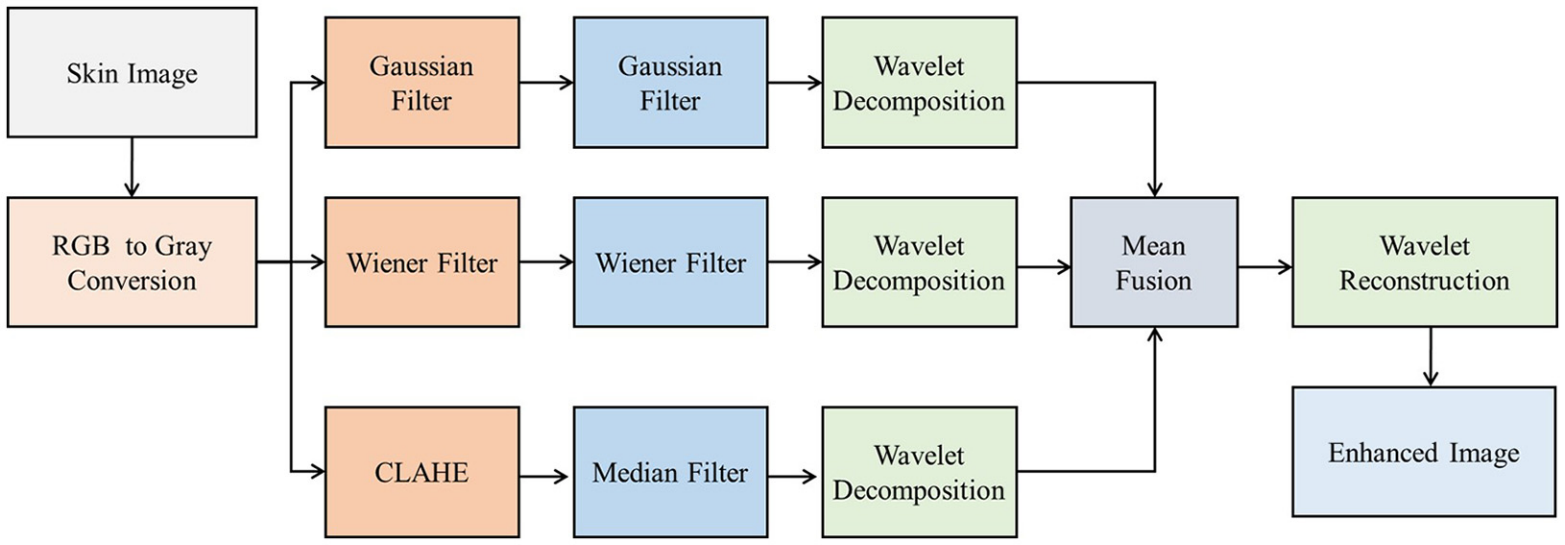
Improved contrast, text, and edge-aware filtering (ICTEF) for skin image enhancement.

The flow diagram of skin image enhancement using the improved contrast, text, and edge-aware filtering (ICTEF) technique, which includes double-stage Gaussian, Wiener filtering, and median filtering, is shown in Figure 3. Gaussian filtering is applied for texture smoothing and reducing high-frequency noise. Wiener filtering is used to minimize blur and noise. Contrast-limited adaptive histogram equalization enhances contrast, and the median filter smooths the skin image's texture. The outputs of the three arms are decomposed using the discrete wavelet transform (DWT) to retain the edge information and low-frequency details of the images. Furthermore, mean fusion is applied to each decomposed component of the three arms of the filters. The inverse DWT (IDWT) is used to reconstruct the image, resulting in an enhanced image.

Here, the Gaussian filter acts as a low-pass filter, removing high-frequency components in the image that cause irregularity in the image texture, as shown in Equation 1.


$$\begin{array}{l} G\left(x,y\right)=\frac{1}{2\pi \sigma^{2}}e^{-\frac{x^{2}+y^{2}}{2\sigma^{2}}} \end{array}$$ (1)

where σ denotes the Gaussian distribution's standard deviation, and *x* and *y* represent the positions of pixels within the Gaussian window. The DWT decomposition of the double-stage Gaussian filtered image, the Wiener filtered image, and the median filtered image is depicted using Equations 2–4, respectively.


$$\begin{array}{l} DWT\left(I_{G}\right)={\left\{A_{G}^{j},V_{G}^{j},H_{G}^{j},D_{G}^{j}\right\}}_{j=1}^{J} \end{array}$$ (2)

$$\begin{array}{l} DWT\left(I_{W}\right)={\left\{A_{W}^{j},V_{W}^{j},H_{W}^{j},D_{W}^{j}\right\}}_{j=1}^{J} \end{array}$$ (3)

$$\begin{array}{l} DWT\left(I_{M}\right)={\left\{A_{M}^{j},V_{M}^{j},H_{M}^{j},D_{M}^{j}\right\}}_{j=1}^{J} \end{array}$$ (4)

Here, *A*, *V*, *H*, and *D* represent the approximation, vertical, horizontal, and diagonal components of the DWT decomposition, respectively. *I*_*G*_, *I*_*W*_, and *I*_*M*_ denote the Gaussian-filtered image, Wiener-filtered image, and median-filtered image, while *J* indicates the number of DWT decomposition levels. The mean of approximation, horizontal, vertical, and diagonal components are given in Equations 5–8, respectively.


$$\begin{array}{l} A_{m}=\frac{AG_{J}+AW_{J}+AM_{J}}{3} \end{array}$$ (5)

$$\begin{array}{l} H_{m}=\frac{HG_{J}+HW_{J}+HM_{J}}{3} \end{array}$$ (6)

$$\begin{array}{l} V_{m}=\frac{VG_{J}+VW_{J}+VM_{J}}{3} \end{array}$$ (7)

$$\begin{array}{l} D_{m}=\frac{DG_{J}+DW_{J}+DM_{J}}{3} \end{array}$$ (8)

Here, *A*_*m*_, *V*_*m*_, *H*_*m*_, and *D*_*m*_ symbolize the mean approximation, mean vertical, mean horizontal, and mean diagonal components of the skin image. The enhanced image is obtained by using the inverse DWT of the mean components of the three arms, which helps to retain the edge information in the fused image as given in Equation 9.


$$\begin{array}{l} \text{Enhanced\_Image}=IDWT\left(A_{m},V_{m},H_{m},D_{m}\right) \end{array}$$ (9)

For the pre-processing stage, we used a three-level DWT decomposition, a 3 × 3 window for median filtering, a 3 × 3 window for Gaussian filtering with a standard deviation of 1, and the db2 wavelet for DWT decomposition.

### 4.2 GLCM features

GLCM is a statistical method for extracting texture features based on the pixel frequency within the spatial dimensions of an image's gray-level co-occurrence matrix. Although GLCM identifies 12 features, the proposed work focuses on essential features for texture analysis, including contrast, correlation, energy, homogeneity, autocorrelation, and entropy.

**Figure 4:**
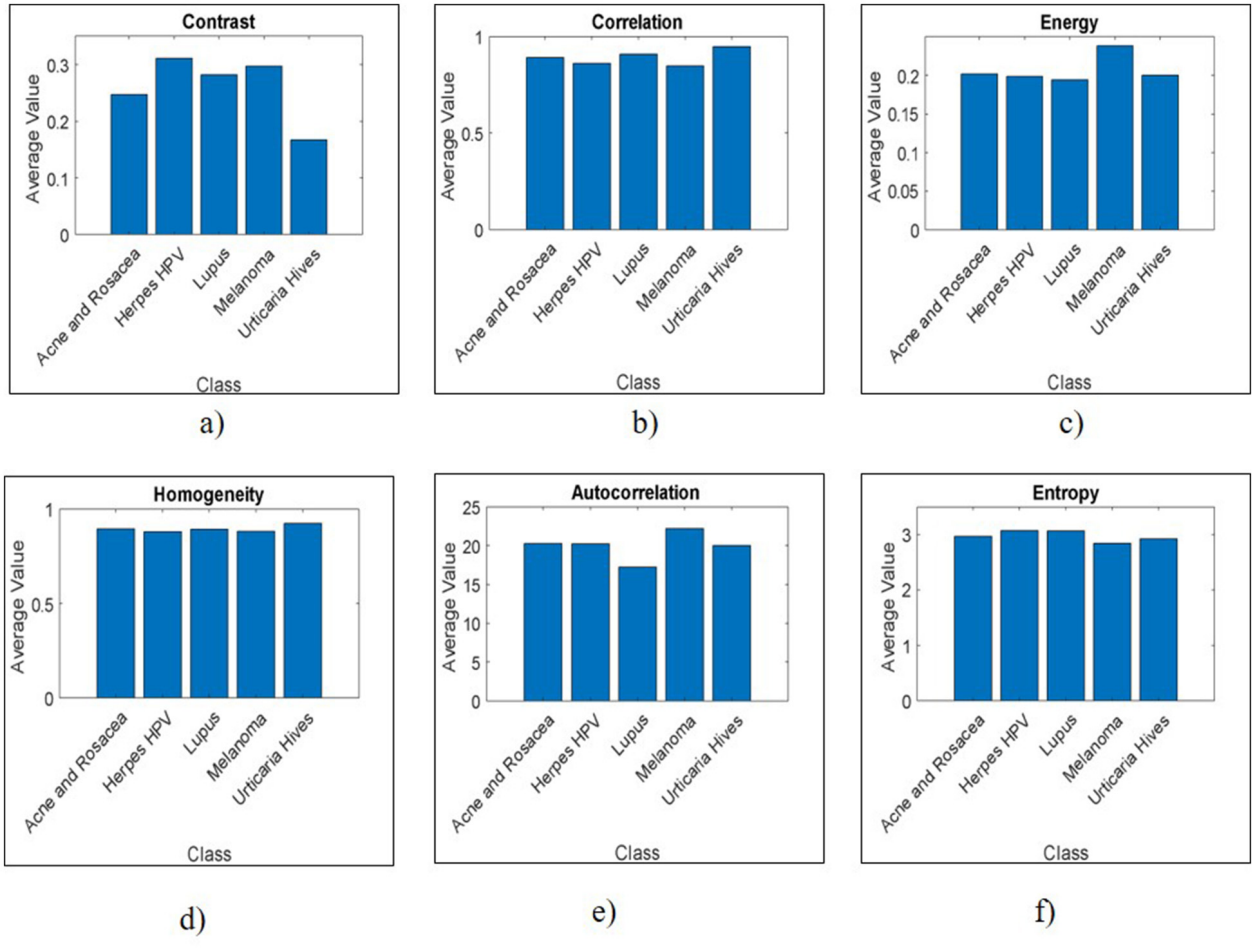
Visualizations of GLCM features: **(a)** Contrast, **(b)** Correlation, **(c)** Energy, **(d)** Homogeneity, **(e)** Autocorrelation, **(f)** Entropy.

- Contrast: Contrast (CT) measures the intensity variations in skin images to illustrate structural changes, as shown in Equation 10. Herpes HPV (0.311) and Melanoma (0.298) have the highest contrast, indicating greater intensity variations and more textured patterns. Conversely, Urticaria Hives (0.167) has the lowest contrast, suggesting a smoother and more uniform texture as shown in Figure 4a.

$$\begin{array}{l} CT=\overset{N-1}{\underset{i,j=0}{\sum }}{\left(i-j\right)}^{2}P_{ij} \end{array}$$ (10)

The CT value ranges from 0 for non-homogeneous regions of diseased images to closer to 0 for normal skin images.

- Correlation: Correlation (CR) represents the similarity between different pixels in the image, with values ranging from –1 to 1, as indicated in Equation 11. Urticaria Hives (0.948) shows the highest correlation, reflecting a strong linear relationship between neighboring pixel intensities. Melanoma (0.848) has the lowest correlation, indicating less consistent pixel relationships, likely due to more irregular or heterogeneous textures as given in Figure 4b.

$$\begin{array}{l} CR=\overset{N-1}{\underset{i,j=0}{\sum }}P_{ij}\frac{\left(i-\mu_{i}\right)\left(j-\mu_{j}\right)}{\sigma^{2}} \end{array}$$ (11)

Where μ_*i*_ denotes the mean over rows, μ_*j*_ symbolizes the mean over columns, and σ describes the variance over columns and rows of skin disease images.

- Energy: Energy (EN) is the squared sum of the GLCM values, indicating uniformity in skin texture, calculated using Equation 12. EN values range from 0 to 1. Melanoma (0.238) has the highest energy, signifying higher textural uniformity or repeated patterns. Lupus (0.194) has the lowest, reflecting more textural complexity or randomness in textures as given in Figure 4c.

$$\begin{array}{l} EN=\overset{N-1}{\underset{i,j=0}{\sum }}{\left(P_{ij}\right)}^{2} \end{array}$$ (12)

- Homogeneity: Homogeneity (HM) describes the closeness of GLCM values to its diagonal elements, with a value of 0 for non-diagonal elements and 1 for diagonal elements, as shown in Equation 13. Urticaria Hives (0.924) and Acne (0.895) rank highest in homogeneity, indicating smoother transitions in gray levels, suggesting these diseases tend to present in visually smoother patterns. Herpes HPV (0.879) is the lowest, aligning with its higher contrast as shown in Figure 4d.

$$\begin{array}{l} HM=\overset{N-1}{\underset{i,j=0}{\sum }}\frac{P_{ij}}{1+|i-j|} \end{array}$$ (13)

- Autocorrelation: Autocorrelation (AC) provides the periodic pattern similarity in skin images, as given by Equation 14. Melanoma (22.19) shows the highest autocorrelation, indicating large, consistent patterns. Lupus (17.22) is the lowest, suggesting more scattered or disrupted texture as shown in Figure 4e.

$$\begin{array}{l} \text{Autocorrelation}=\overset{N-1}{\underset{i,j=0}{\sum }}P_{ij}\cdot \left(i\cdot j\right) \end{array}$$ (14)

- Entropy: Entropy (ET) measures the information in the image and provides a randomness measure using Equation 15. Herpes HPV (3.08) and Lupus (3.07) show higher entropy, suggesting more randomness and disorder in texture. Melanoma (2.85) and Urticaria Hives (2.93) exhibit lower entropy, reflecting more orderly textures like in Figure 4f

$$\begin{array}{l} \text{Entropy}=\overset{N-1}{\underset{i,j=0}{\sum }}(-P_{ij}\ln\left(P_{ij}\right)) \end{array}$$ (15)

The final GLCM feature *Feat*_*GLCM*_ is formed by combining GLCM features as given in Equation 16.


$$\begin{array}{l} {\text{Feat}}_{GLCM}=\left\{CT,CR,EN,HM,AC,ET\right\} \end{array}$$ (16)

### 4.3 Improved LBP features

LBP provides scale- and rotation-invariant texture features for skin images. LBP helps detect local fine changes in skin texture, which can be used to characterize skin diseases. LBP considers the local 3 × 3 window, comparing neighboring pixels with the centered pixel. If the neighboring pixel's value is greater than the centered pixel, it is considered binary 1; if less, it is binary 0. The neighboring pixels provide eight bits for binary values, which are then converted into a decimal value. The centered value of the window is replaced by this decimal value as given in Equation 17. Further, the histogram of the LBP descriptor is computed over N × N block.


$$LBP(x,y)=\{\begin{array}{ll} 1, & \text{if }im(x,y)>c(x,y)\\ 0, & \text{otherwise} \end{array}$$ (17)

**Figure 5:**
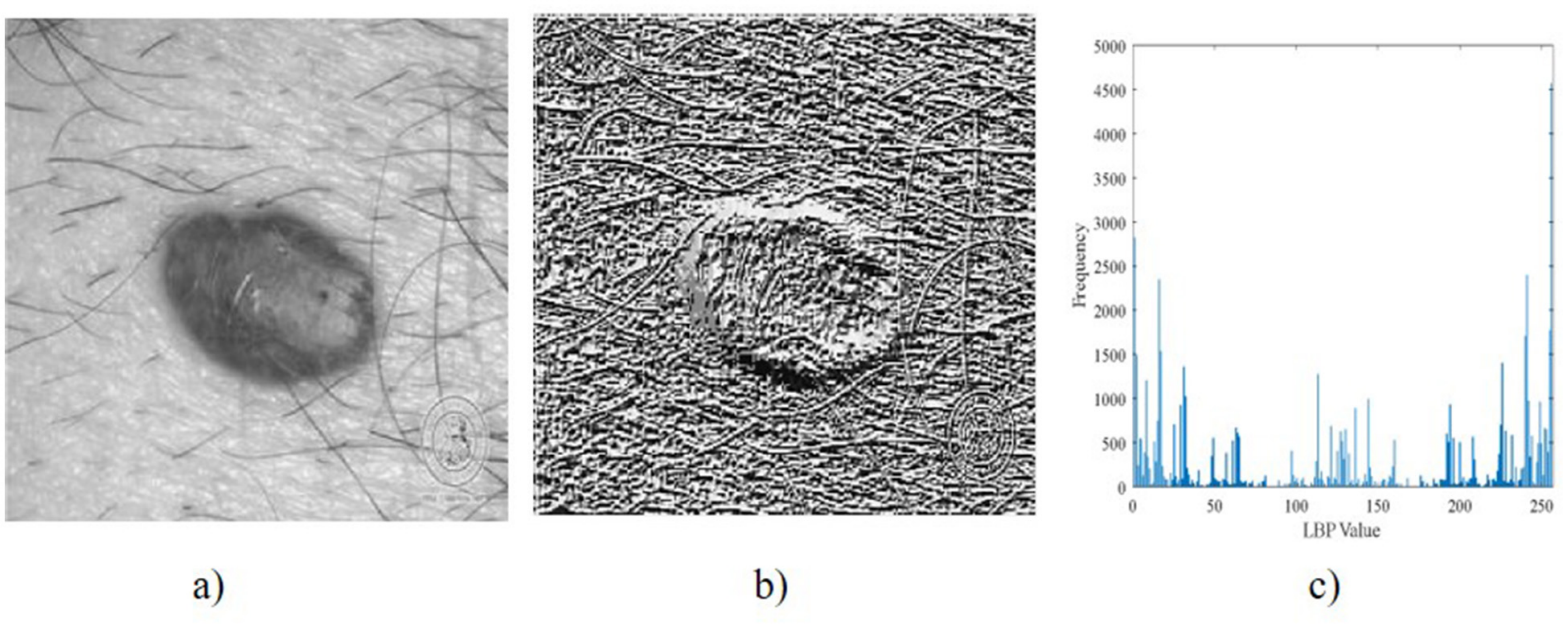
LBP visualizations: **(a)** Gray skin image, **(b)** LBP descriptor, **(c)** LBP histogram.

The visualizations of the LBP texture descriptor and LBP histograms are shown in Figure 5.

**Figure 6:**
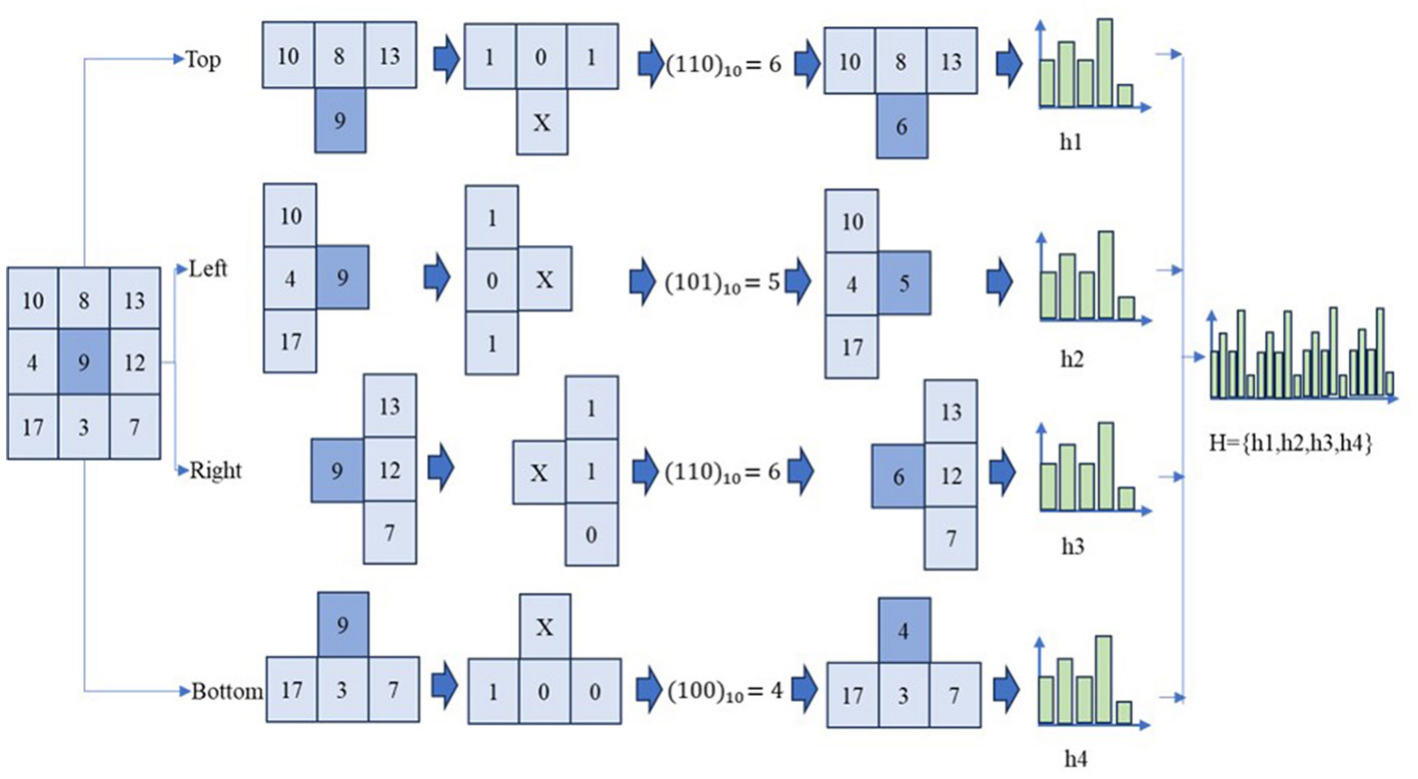
Improved LBP process.

The conventional LBP method offers limited spatial connectivity for skin disease images by evaluating the relationship between all directional neighbors and the central pixels.The newly enhanced LBP improves the spatial connectivity of pixels in skin images by analyzing four separate directions, which helps in reducing LBP features and consequently decreases computational demands. This improved LBP (ILBP) divides the local window into four sections—top, left, right, and bottom—to establish the spatial relationship of skin images in four dimensions as illustrated in Figure 6. Similar to the traditional LBP, neighboring pixels are compared with the center pixel, resulting in a binary pattern with three possible values (000–111). The decimal equivalent of this binary pattern is then placed at the center pixel, forming four descriptors with a total of 8 possible values ranging from 0 to 7. The histograms from the top, left, bottom, and right are combined, yielding a total of 32 LBP features, which is fewer than the 256 features of the traditional LBP.

Let *im*(*x, y*) denote a grayscale skin image. For each pixel location (*x, y*), a 3 × 3 local neighborhood is given by Equation 18.


$$\begin{array}{l} \text{W}\left(x,y\right)=\left[\begin{array}{ccc} p_{1} & p_{2} & p_{3}\\ p_{4} & c & p_{5}\\ p_{6} & p_{7} & p_{8} \end{array}\right] \end{array}$$ (18)

where *c* represents the center pixel of local window and *p*_*i*_, *i* = 1, …, 8 denote its neighboring pixels. The 3 × 3 window is partitioned into four directional patterns for top U, bottom B, left L, and right R, each sharing the common center pixel *p*_*c*_, as defined using Equations 19–22 which yields texture descriptors in the four directions as ${\text{LBP}}_{U}$, ${\text{LBP}}_{B}$, ${\text{LBP}}_{L}$, and ${\text{LBP}}_{R}$.


$$\begin{array}{l} {\text{LBP}}_{U}=\left\{p_{1},p_{2},p_{3},p_{c}\right\} \end{array}$$ (19)

$$\begin{array}{l} {\text{LBP}}_{B}=\left\{p_{6},p_{7},p_{8},p_{c}\right\} \end{array}$$ (20)

$$\begin{array}{l} {\text{LBP}}_{L}=\left\{p_{1},p_{4},p_{6},p_{c}\right\} \end{array}$$ (21)

$$\begin{array}{l} {\text{LBP}}_{R}=\left\{p_{3},p_{5},p_{8},p_{c}\right\} \end{array}$$ (22)

For each pattern $q\in \left\{{\text{LBP}}_{U},{\text{LBP}}_{B},{\text{LBP}}_{L},{\text{LBP}}_{R}\right\}$, a histogram is computed to provide shift invariance and rotation invariance using Equation 23, where 1(·) denotes the indicator function.


$$\begin{array}{l} H_{q}\left(b\right)=\underset{x,y}{\sum }1({\text{LBP}}_{q}\left(x,y\right)=b),\;b=0,1,\ldots ,2^{3}-1 \end{array}$$ (23)

The final ILBP feature vector is obtained by concatenating the histograms of all four patterns as given in Equation 24.


$$\begin{array}{l} {\text{F}}_{\text{ILBP}}=\left[H_{U},\;H_{B},\;H_{L},\;H_{R}\right] \end{array}$$ (24)

### 4.4 HOG features

**Figure 7:**
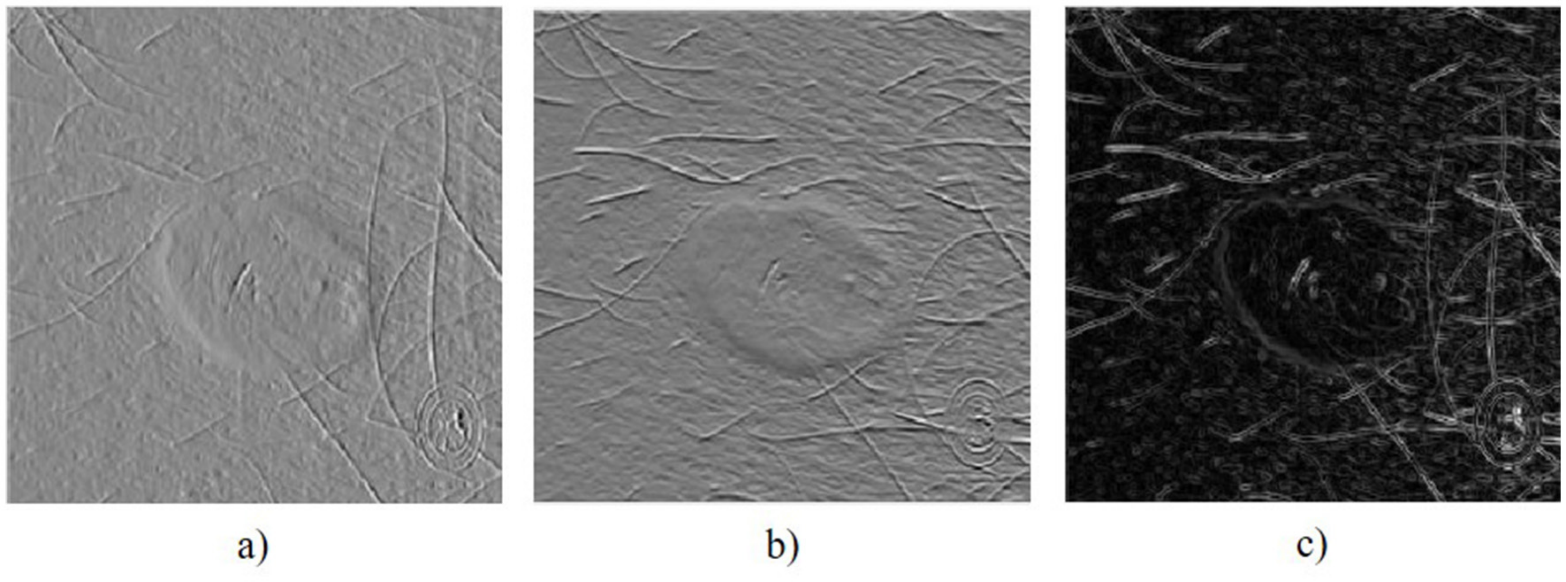
HOG visualization: **(a)** Horizontal gradients, **(b)** Vertical gradient, **(c)** Magnitude of gradient.

HOG features capture the shape characteristics of the skin image to identify changes in skin lesions. HOG is calculated over a cell size of 8 × 8 pixels, a block size of 2 × 2 cells with 50% overlap, and orientations are determined over nine bins. The HOG feature extraction process includes image normalization, horizontal and vertical gradient computation, and orientation calculation over the bins to define shape features, as depicted in Figure 7. Horizontal and vertical gradients are computed using horizontal (Hx) and vertical (Hy) kernels, as specified in Equations 25–28.


$$\begin{array}{l} H_{x}=\left[-1\;0\;1\right] \end{array}$$ (25)

$$\begin{array}{l} H_{y}={\left[-1\;0\;1\right]}^{T} \end{array}$$ (26)

$$\begin{array}{l} I_{x}=I*H_{x} \end{array}$$ (27)

$$\begin{array}{l} I_{y}=I*H_{y} \end{array}$$ (28)

The gradient magnitude is given by Equation 29, and the orientation is described by Equation 30. Orientations are calculated over nine bins for a single cell, and a block provides 36 orientations, resulting in a total of 34,596 HOG features for a skin image with a resolution of 256 × 256 pixels. HOG features describe the shape of skin objects, highlighting irregularities in perimeter, shape, and size of the object.


$$\begin{array}{l} M=\sqrt{I_{x}^{2}+I_{y}^{2}} \end{array}$$ (29)

$$\begin{array}{l} \theta =\tan^{-1}\left(\frac{I_{y}}{I_{x}}\right) \end{array}$$ (30)

### 4.5 SDNet framework

The SDNet includes parallel 2D-CNN and 1D-CNN models that process the pre-processed image and a combination of GLCM, LBP, and HOG features as input. The 2D-CNN comprises three convolutional layers with 64, 128, and 256 filters, followed by a rectified linear unit (ReLU) layer and a batch normalization (BN) layer. These convolution layers learn the spatial connectivity and correlation between local regions of skin images. The 1D-CNN takes a handcrafted feature vector that combines GLCM, improved LBP, and HOG features to describe the texture and shape attributes of skin images. The maximum pooling layer (MaxPool) are applied to select the salient feature and minimize the deep feature dimensions. MaxPool layer helps to improve training speed by minimizing network computational complexity. The deep features from the last maxPool layer of both 2D-CNN and 1D-CNN are flattened and fed into the fully connected (FC) layer to enhance feature connectivity. Finally, a softmax classifier is used to categorize the skin images into normal and diseased classes. The hyperparameters used for the SDNet are listed in Table 1 and the configurations of different layers along with trainable paramters is provided in Table 2.

**Table 1:** Hyper-parameters of SDNet.

**Parameter**	**Specification**
Learning algorithm	Adam
Decay rate	0.5
Epochs	200
Learning rate	0.001
Train–Test split ratio	70:30

**Table 2:** Detailed architecture and trainable parameters of the proposed model.

**Model**	**Sublayer**	**Input size**	**No. of filters/units**	**Filter size**	**Stride**	**Output size**	**Trainable parameters**
2D-DCNN	Input image	256 × 256 × 3	–	–	–	256 × 256 × 3	0
	Conv2D	256 × 256 × 3	64	3 × 3	1	256 × 256 × 64	1,792
	ReLU	256 × 256 × 64	–	–	–	256 × 256 × 64	0
	BatchNorm	256 × 256 × 64	–	–	–	256 × 256 × 64	128
	MaxPool	256 × 256 × 64	–	2 × 2	2	128 × 128 × 64	0
	Conv2D	128 × 128 × 64	128	3 × 3	1	128 × 128 × 128	73,856
	ReLU	128 × 128 × 128	–	–	–	128 × 128 × 128	0
	BatchNorm	128 × 128 × 128	–	–	–	128 × 128 × 128	256
	MaxPool	128 × 128 × 128	–	2 × 2	2	64 × 64 × 128	0
	Conv2D	64 × 64 × 128	256	3 × 3	1	64 × 64 × 256	295,168
	ReLU	64 × 64 × 256	–	–	–	64 × 64 × 256	0
	BatchNorm	64 × 64 × 256	–	–	–	64 × 64 × 256	512
	MaxPool	64 × 64 × 256	–	2 × 2	2	32 × 32 × 256	0
	Flatten	32 × 32 × 256	–	–	–	262,144	0
1D-DCNN	Input features	34, 596 × 1	–	–	–	34, 596 × 1	0
	Conv1D	34, 596 × 1	64	1 × 3	1	34, 596 × 64	256
	ReLU	34, 596 × 64	–	–	–	34, 596 × 64	0
	BatchNorm	34, 596 × 64	–	–	–	34, 596 × 64	128
	MaxPool	34, 596 × 64	–	1 × 2	2	17, 298 × 64	0
	Conv1D	17, 298 × 64	128	1 × 3	1	17, 298 × 128	512
	ReLU	17, 298 × 128	–	–	–	17, 298 × 128	0
	BatchNorm	17, 298 × 128	–	–	–	17, 298 × 128	256
	MaxPool	17, 298 × 128	–	1 × 2	2	8, 649 × 128	0
	Conv1D	8, 649 × 128	256	1 × 3	1	8, 649 × 256	1,024
	ReLU	8, 649 × 256	–	–	–	8, 649 × 256	0
	BatchNorm	8, 649 × 256	–	–	–	8, 649 × 256	512
	MaxPool	8, 649 × 256	–	1 × 2	2	4, 324 × 256	0
	Flatten	4, 324 × 256	–	–	–	1,106,944	0
Fusion	Concatenation	262, 144+1, 106, 944	–	–	–	1,369,088	0
FC layer	Fully connected	1,369,088	4	–	–	4	5,476,352
Classifier	Softmax	4	4	–	–	4	0
Total	Trainable parameters	–	–	–	–	–	**5,850,752**

The bold value indicates the total number of trainable parameters of the proposed SDNet.

## 5 Results and discussions

The proposed system is implemented using MATLAB R2024b on a personal computer system having 16 GB RAM, a core i5 processor, and 4 GB graphics.

### 5.1 Dataset

The publicly accessible DermNet dataset (DermNet dataset, 2024) is utilized to assess the performance of SDNet across five skin disorders. These disorders include acne (840 samples), lupus (420 samples), melanoma (463 samples), urticaria hives (375 samples), and herpes HPV (405 samples).

### 5.2 Evaluation metrics

The effectiveness of the proposed SDNet is measured using accuracy (AC), recall (REC), precision (PREC), and F1-score (F1S). Accuracy reflects the system's overall correctness, recall provides a quantitative assessment, and precision offers another quantitative measure of the system. The F1-score represents the harmonic mean of recall and precision. The AC, REC, PREC, and F1S are calculated using Equations 31–34, where nTPV, nTNV, nFPV, and nFNV denote the true positive count, true negative count, false positive count, and false negative count, respectively.


$$\begin{array}{l} A=\frac{nTPV+nTNV}{nTPV+nTNV+nFPV+nFNV} \end{array}$$ (31)

$$\begin{array}{l} \text{PREC}=\frac{nTPV}{nTPV+nFPV} \end{array}$$ (32)

$$\begin{array}{l} \text{REC}=\frac{nTPV}{nTPV+nFNV} \end{array}$$ (33)

$$\begin{array}{l} F1S=\frac{2\times \text{PREC}\times \text{REC}}{\text{PREC}+\text{REC}} \end{array}$$ (34)

### 5.3 Discussions on the results

**Figure 8:**
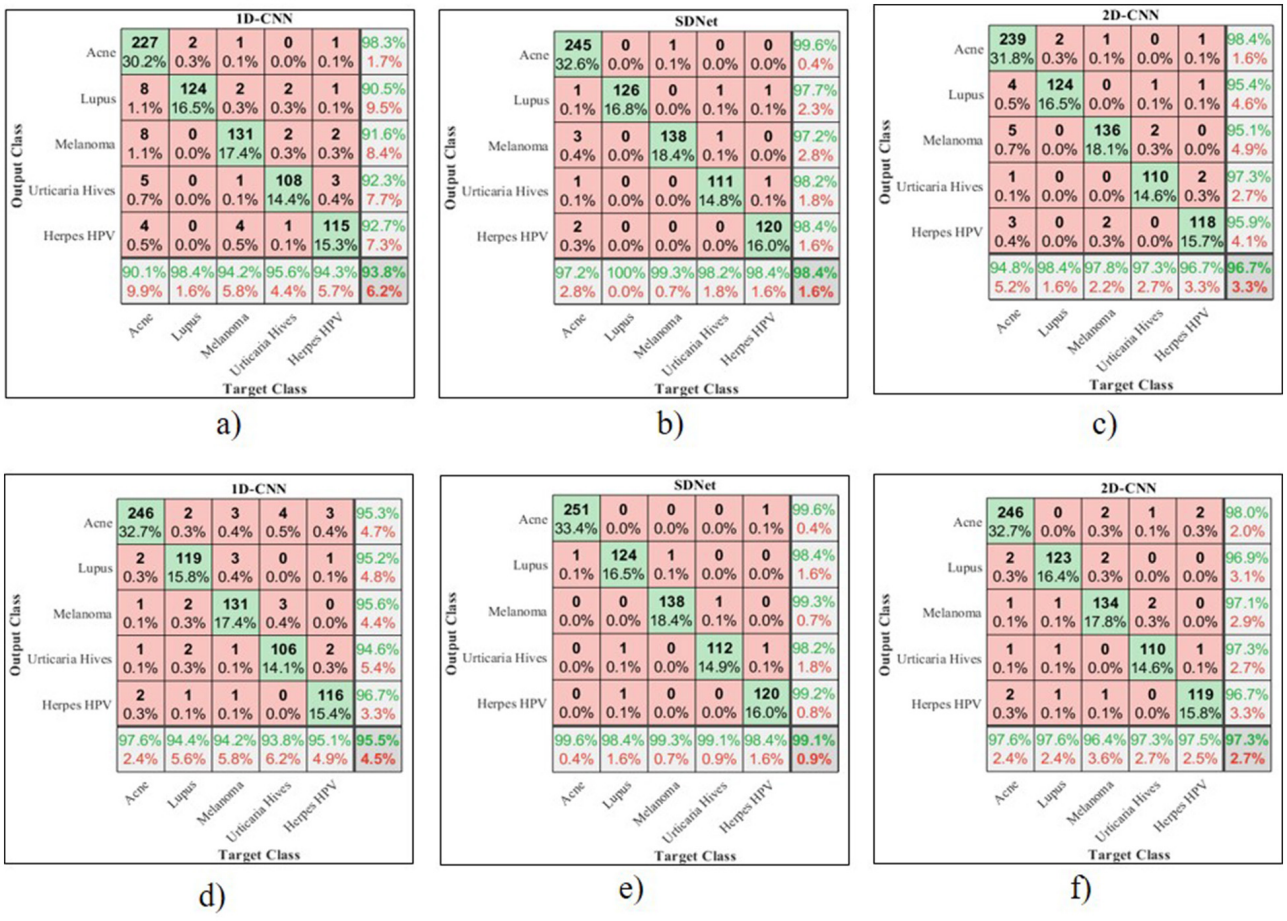
Confusion matrix for **(a)** 1D-CNN, **(b)** 2D-CNN, **(c)** SDNet without pre-processing, **(d)** 1D-CNN, **(e)** 2D-CNN, **(f)** SDNet with pre-processing.

The confusion matrix for 2D-CNN, 1D-CNN, and SDNet is shown in Figure 8.

**Figure 9:**
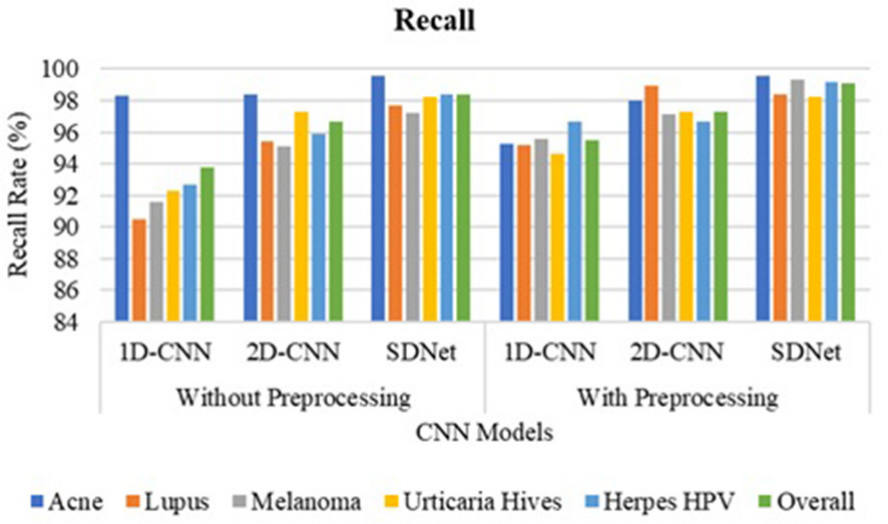
Recall comparison of SDNet.

**Figure 10:**
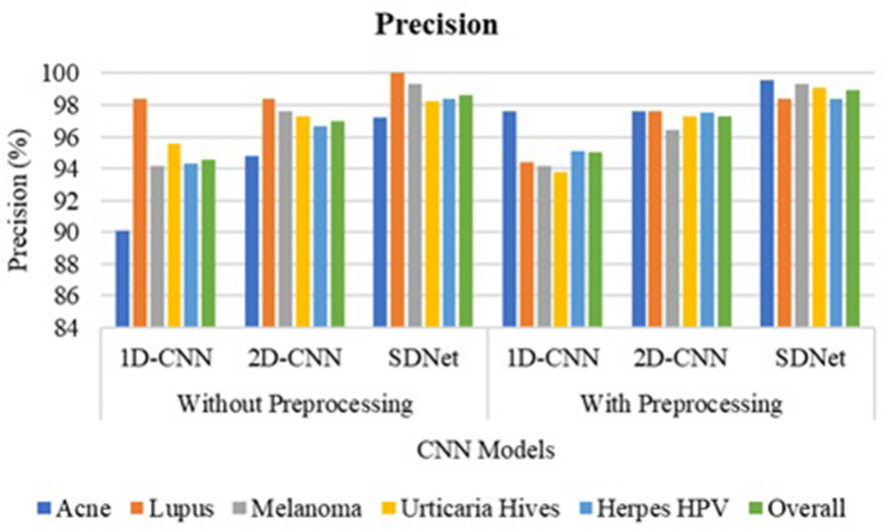
Precision comparison of SDNet.

**Figure 11:**
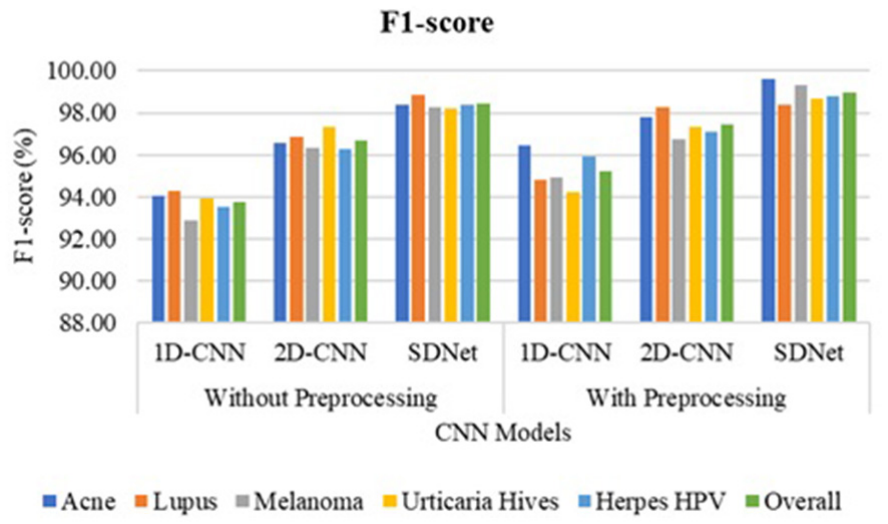
F1-score comparison of SDNet.

**Figure 12:**
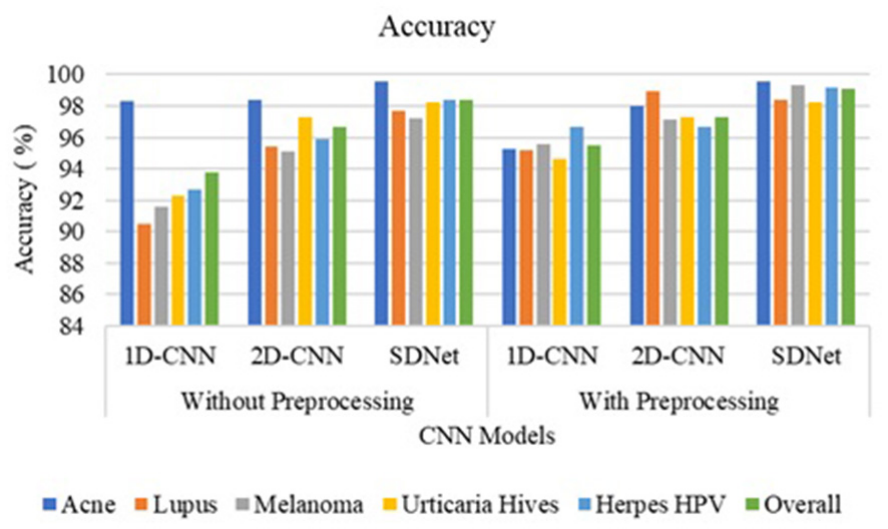
Accuracy comparison of SDNet.

The 1D-CNN achieves an overall accuracy of 93.8% using GLCM, LBP, and HOG features; the 2D-CNN achieves 96.7% using original skin images; and the proposed SDNet achieves an improved overall accuracy of 98.45% without pre-processing. Noise, blur, and irregularities in skin images can impede the learning of distinctive features using CNN. However, with pre-processing, the 1D-CNN, 2D-CNN, and SDNet achieve overall accuracies of 95.5%, 97.3%, and 99.1%, respectively. The analysis of skin disease detection models under two scenarios—without and with image pre-processing—demonstrates the significant impact of pre-processing on classification accuracy and other evaluation metrics such as precision, recall, and F1-score, as shown in Figures 9–12, respectively.

Three deep learning models, namely 1D-CNN, 2D-CNN, and SDNet, were tested on five different skin diseases: Acne, Lupus, Melanoma, Urticaria Hives, and Herpes HPV. Without pre-processing, the models relied on raw, unprocessed images for classification. The 1D-CNN model recorded an overall accuracy of 93.8%, precision of 94.52%, recall of 93.8%, and F1-score of 93.72%. The 2D-CNN model showed better performance, achieving an accuracy of 96.7%, a precision of 96.96%, a recall of 96.7%, and an F1-score of 96.68%. Among all, SDNet performed the best even without pre-processing, attaining 98.4% accuracy, 98.62% precision, 98.4% recall, and 98.41% F1-score. After applying image pre-processing techniques such as contrast enhancement and noise reduction, a noticeable improvement was observed across all models and all performance measures. The 1D-CNN model's overall accuracy increased from 93.8% to 95.5%, reflecting an improvement of approximately 1.7%. Its precision improved by 0.5% (from 94.52% to 95.02%), recall by 1.7%, and F1-score by 1.5%. Similarly, 2D-CNN saw its accuracy increase from 96.7% to 97.3%, resulting in a 0.6% improvement. Its precision increased by 0.32%, recall by 0.6%, and F1-score by 0.76%. The SDNet with pre-processing provides the highest recall of 99.6% for acne and the lowest recall of 98.2% for urticaria hives. In contrast, SDNet without pre-processing offers the highest recall of 98% for acne and the lowest recall of 97.3% for urticaria hives. Similarly, the SDNet with pre-processing offers the highest improved precision of 99.6% for acne compared to the SDNet without pre-processing (97.6%). The most notable improvement was observed in the SDNet model, where accuracy increased from 98.4% to 99.1%, representing a 0.7% improvement. Precision increased from 98.62% to 98.96%, recall from 98.4% to 99.1% (0.7% gain), and F1-score from 98.41% to 98.95%, a 0.54% enhancement.

In terms of disease-specific improvements, the most notable percentage gains after pre-processing were seen in Lupus and Melanoma, particularly with the 1D-CNN model. For Lupus, the recall rate of the 1D-CNN rose by 4.7%, moving from 90.5% to 95.2%, while for Melanoma, it increased by 4%, from 91.6% to 95.6%. Similarly, Herpes HPV saw a 4% improvement in recall with the 1D-CNN. Although SDNet already had a high baseline performance, pre-processing still led to small but consistent enhancements across all diseases, notably pushing recall and F1-scores above 99% for most categories. Table 3 compares the accuracy of disease detection across various feature representations and algorithms. The 1-D CNN achieves an overall accuracy of 78.35% using six GLCM features, 86.40% with 256 LBP features, 88.20% for 32 ILBP, and 90.15% for 34,596 HOG features in skin disease detection, based on an image size of 256 × 256 pixels. Combining different features enhances the distinctiveness of skin texture, resulting in improved accuracy of 91.75% for GLCM+ILBP, 93.10% for GLCM+HOG, 93.20% for ILBP+HOG, and 95.5% for GLCM+ILBP+HOG. The 2D-CNN achieves an overall accuracy of 97.3% for original images by learning spatial correlations and connections in skin images. The proposed two-way feature depiction takes advantage of both traditional and original image features, offering superior attention between deep features, which leads to an overall improved accuracy of 99.1% for skin disease detection.

**Table 3:** Performance comparison for different features.

**Features**	**Classifiers**	**Accuracy (%)**
GLCM	1D-CNN	78.35
LBP	1D-CNN	86.40
ILBP	1D-CNN	88.20
HOG	1D-CNN	90.15
GLCM + ILBP	1D-CNN	91.75
GLCM + HOG	1D-CNN	93.10
ILBP + HOG	1D-CNN	93.20
GLCM + ILBP + HOG	1D-CNN	95.5
Original image	2D-CNN	97.3
GLCM + ILBP + HOG + Original image	SDNet (1D-CNN + 2D-CNN)	99.1

### 5.4 Grad-CAM

**Figure 13:**
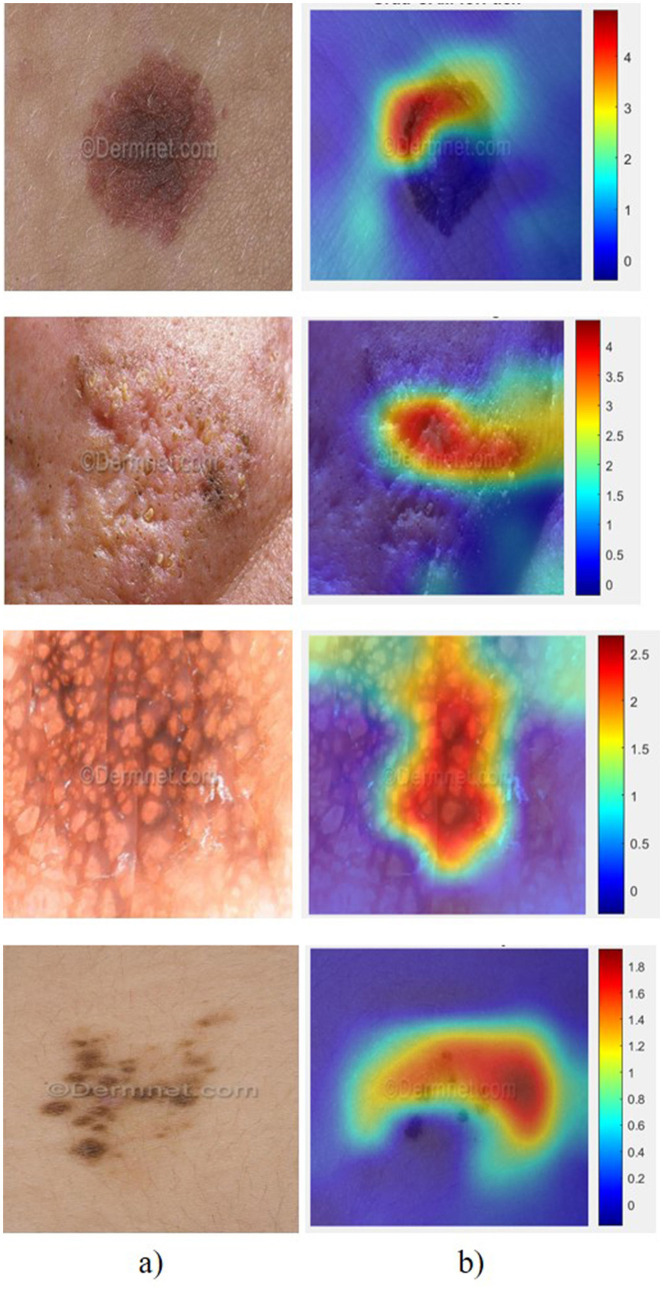
Grad-CAM analysis of several samples from the dataset: **(a)** Original sample and **(b)** Grad-CAM visualization.

To enhance the interpretability of the proposed hybrid 2D–1D DCNN model, Gradient-weighted Class Activation Mapping (Grad-CAM) is employed to visualize the spatial regions that most influence the classification decision. Grad-CAM is applied to the final convolutional layer of the 2D CNN branch, as this layer preserves high-level spatial semantics of the input medical images. The generated heatmaps consistently highlight clinically relevant lesion regions, such as abnormal tissue boundaries and high-density pathological areas, which align with expert-annotated regions of interest. This correspondence indicates that the model learns meaningful disease-related features rather than spurious patterns. While the 1D CNN branch captures discriminative handcrafted feature representations, Grad-CAM operates exclusively on the image-based branch to provide spatial explainability. The Grad-CAM output does not alter the prediction pipeline but serves as a *post-hoc* interpretability module, thereby improving clinical trust and transparency of the proposed hybrid architecture. Figure 13 illustrates the Grad-CAM analysis of images based on the SDNet model for some sample images. Grad-CAM provides insights into how the feature maps of the original image content influence disease prediction. We have These areas usually correspond to critical features like lesions, discolorations, or unusual textures that the model identifies as potential disease indicators. Cooler colors, such as blue, denote regions with minimal or no impact on the prediction. This visual explanation allows clinicians to verify whether the model is focusing on medically relevant areas or being distracted by irrelevant background features. Grad-CAM not only aids in model validation and trust-building but also supports medical professionals in making informed decisions by highlighting potentially overlooked regions. Although Grad-CAM analysis is typically applied to pure CNN models, in the proposed approach, mapping Grad-CAM heatmaps against regions with high handcrafted feature values can confirm that the CNN focuses on clinically relevant areas. This enhances trust and explainability for dermatologists by correlating heatmap hotspots with known dermatological markers.

The system's results are compared with previous techniques for five-class skin disease detection using the DermNet dataset, as shown in Table 4. We have implemented VGGNet, AlexNet, GoogleNet, EfficientNet, ConvNextV2, VisionTransfromer and an ensemble classifier (AlexNet, VGGNet, and and GoogleNet) as described in (Mandache et al. 2018) as well as CNN-SVM, for the pre-processed dataset in five-class disease detection. The proposed SDNet demonstrates an enhanced accuracy of 99.10%, surpassing VGGNet's 89.25%, AlexNet's 90.95%, GoogleNet's 91.25%, the ensemble's 93.50%, CNN-SVM's 93.80%, EfficientNet's 94.25%, ConvNextV2's 94.25%, and VisionTransformer's 96.15%. Traditional transfer learning methods typically use only images as input, which can sometimes overlook subtle details in the skin image's orientation and texture. SDNet, however, integrates the texture, orientation, and shape characteristics of the skin image. The VisionTransformer has shown good accuracy but requires a longer training time of 4,532 s due to its complex architecture. This lightweight framework requires less training time, taking 2,341 s, and a detection time of 0.93 s for identifying five classes of skin diseases, outperforming conventional algorithms.

**Table 4:** Comparison of different algorithms in terms of accuracy, training time, and detection time.

**Algorithm**	**Accuracy (%)**	**Training time (sec)**	**Detection time (sec)**
VGG16 (Mandache et al., 2018)	89.25	3,134	1.23
AlexNet (Mandache et al., 2018)	90.95	3,056	1.07
GoogleNet (Mandache et al., 2018)	91.25	3,678	1.47
Ensemble classifier (Mandache et al., 2018)	93.50	3,427	1.34
CNN-SVM (Mandache et al., 2018)	93.80	2,765	1.03
EfficientNet (Sharma, 2025)	94.25	2,845	1.23
ConvNextV2 (Woo et al., 2023)	94.20	2,753	1.17
VisionTransformer (Wang et al., 2025)	96.15	4,532	1.64
Proposed SDNet	99.1	2,341	0.93

We have analyzed the system's performance using 5-fold and 10-fold cross-validation to assess stability and the statistical properties of the results, as shown in Table 5. It achieves a mean accuracy of 97.55% in 5-fold and 98.75% in 10-fold cross-validation for skin disease detection. The lower standard deviation of 0.32 provides superior consistency in results in a 10-fold cross-validation than a 5-fold cross-validation for skin disease detection. The 10-fold cross-validation offers a narrower confidence interval (CI) of [98.49, 99.10] than [96.73, 98.37] of 5-fold cross-validation, which shows the consistency and reliability in the skin disease detection results.

**Table 5:** Performance comparison using cross-validation.

**Performance metrics**	**5-Fold**	**10-Fold**
Mean accuracy (%)	97.55	98.75
Standard deviation	0.58	0.32
95% Confidence interval	[96.73, 98.37]	[98.49, 99.10]

A limitation of this study is that the proposed model is evaluated using only a single dataset (DermNet). Although the results demonstrate strong performance, the absence of external or cross-dataset validation may limit the generalizability of the model across diverse imaging conditions and populations. Future work will focus on validating the proposed framework on multiple publicly available datasets and performing cross-dataset evaluations to further assess its robustness and clinical applicability.

## 6 Conclusion and future scope

This study introduces a two-way feature representation for skin disease detection, combining 2D-CNN and 1D-CNN. The 2D-CNN processes the original images to learn the correlation and connectivity within the skin pattern, while the 1D-CNN handles various texture and shape features, such as GLCM, ILBP, and HOG, to define the global texture, local texture, and shape attributes of the skin disease image. ILBP offers a superior texture description compared to traditional LBP, enhancing texture spatial connectivity and correlation while reducing features. The novel improved contrast, text, and edge-aware filtering (ICTEF) technique, utilizing double-stage Gaussian, Wiener, and median filtering, enhances edges and texture while reducing noise and contrast. Without pre-processing, SDNet achieves a recall of 98.4%, a precision of 98.62%, an F1-score of 98.41%, and an accuracy of 98.4%. After pre-processing, SDNet's overall accuracy improves to 99.1%, with a recall of 99.1%, a precision of 98.96%, and an F1-score of 98.95% for five-class skin disease detection. The study supports its findings with an explainable AI framework like Grad-CAM, which accurately maps disease locations, thereby increasing trust and transparency among dermatologists for more reliable decisions by the proposed model. Future improvements could involve fine-tuning the model's hyperparameters. The disparity in the training dataset leads to class imbalance, resulting in discrepancies between qualitative and quantitative results. An effective data augmentation scheme can be proposed in the future to address data scarcity and class imbalance. The system can be implemented on a standalone device to aid dermatologists in screening for skin diseases.

## Data Availability

The original contributions presented in the study are included in the article/supplementary material, further inquiries can be directed to the corresponding author.
